# MicroRNA Novel-m0027-3p Negatively Regulates *Jhamt* Gene and Affects Juvenile Hormone Biosynthesis in *Apis mellifera* Larvae

**DOI:** 10.3390/insects17030288

**Published:** 2026-03-06

**Authors:** Ning Wang, Si-Jia Deng, Chuan-Lian Zhang, Gen-Chao Gan, Zi-Nuo Li, Min Jiang, Yi-Wen Liu, Hao-Dong Zhao, Jia-Run Yang, Jian-Feng Qiu, Rui Guo, Guo-Jun Xu, Da-Fu Chen

**Affiliations:** 1 College of Bee Science and Biomedicine, Fujian Agriculture and Forestry University, Fuzhou 350002, China; 2 Apicultural Research Institute of Jiangxi Province, Nanchang 330052, China; 3 National & Local United Engineering Laboratory of Natural Biotoxin, Fuzhou 350002, China; 4 Apitherapy Research Institute of Fujian Province, Fuzhou 350002, China

**Keywords:** *Apis mellifera*, microRNAs, juvenile hormone, *Jhamt*

## Abstract

Juvenile hormone (JH) is a key regulator of larval development in honeybees. Its biosynthesis involves multiple enzymatic steps and is modulated by a complex regulatory network that includes microRNAs (miRNAs). Juvenile hormone acid methyltransferase (JHAMT) catalyzes the final step in JH synthesis. This study demonstrates that the miRNA novel-m0027-3p negatively regulates the expression of the *Jhamt* gene in *Apis mellifera* larvae (*AmJhamt*), thereby mediating JH biosynthesis. Bioinformatics predictions indicate that novel-m0027-3p potentially targets six hormone-related genes (22 mRNAs), including *AmJhamt*. Dual-luciferase reporter assays and mimics/inhibitor-miRNA feeding confirmed that novel-m0027-3p significantly suppresses the expression of the target gene *AmJhamt*. In vivo experiments showed that larvae fed with the mimics of novel-m0027-3p exhibited decreased JH titers and significantly downregulated expression of JH signaling downstream genes *AmHex70b* and *AmKr-h1*. Conversely, larvae fed with the inhibitors of novel-m0027-3p displayed significantly elevated JH titers and markedly upregulated expression of *AmHex70b* and *AmKr-h1*. Our findings provide experimental evidence for the coupling between miRNAs and hormonal pathways in honeybees.

## Simple Summary

Honeybees play a crucial role in sustaining agricultural production and maintaining ecosystem balance. MicroRNAs (miRNAs) are involved in bee development and caste differentiation by regulating gene transcription and translation. Our findings indicate that hormones act as a key bridge in the miRNA-mediated regulation of honeybee physiology. Specifically, miRNA novel-m0027-3p can directly target juvenile hormone (JH) acid methyl transferase (*Jhamt*), a key gene involved in JH synthesis, thereby modulating JH titers and signaling pathways during larval development. Our study provides insights into how miRNAs regulate hormone pathways to affect honey bee development.

## 1. Introduction

MicroRNAs (miRNAs) are a class of endogenous, non-coding RNAs, approximately 18–25 nucleotides in length, which are widely present in eukaryotes. miRNAs regulate gene transcription and translation by specifically binding to the 3′ untranslated region (3′ UTR), 5′ untranslated region (5′ UTR), or coding sequence (CDS) of target mRNAs [1,2]. Primary miRNAs (pri-miRNAs) are transcribed by RNA polymerase II and then cleaved by the Drosha-DGCR8 complex in the nucleus to form precursor miRNAs (pre-miRNAs). These pre-miRNAs are exported to the cytoplasm via Exportin-5 and further processed by Dicer into mature miRNAs [3]. miRNAs are involved in various biological processes in both animals and plants, including development, immune responses, oxidative stress, and antiviral defense [2,4].

Studies have shown that miRNAs play crucial roles in regulating insect growth, development, reproduction, and metabolism [5]. The 179 differentially expressed miRNAs (DEmiRNAs) were identified across different developmental stages of *Tribolium castaneum*, and knockdown of these DEmiRNAs led to defects in metamorphosis and wing development [6]. In *Aedes aegypti*, miR-277 regulates lipid metabolism by modulating the expression of insulin-like peptide genes *ILP7* and *ILP8* [7]. Additionally, multiple miRNAs such as miR-2 and miR-13a can suppress the expression of *Notch*, thereby affecting ovarian development in the migratory locust, *Locusta migratoria manilensis* [8]. While miRNAs regulate gene expression, they themselves are also subject to regulation by other genes. In *Drosophila melanogaster*, miR-927 influences the larval-to-pupal transition by inhibiting the expression of *Kr-h1*, a downstream transcription factor in the JH signaling pathway. Conversely, JH and its receptor gene Methoprene-tolerant (*Met*) feedback to suppress the expression of miR-927 [9].

The timing of molting and metamorphosis in holometabolous insects is primarily regulated by hormones, including juvenile hormone (JH) and ecdysteroids [10,11]. Ecdysone is synthesized in the prothoracic gland and secreted into the hemolymph, where it is converted to the active hormone 20-hydroxyecdysone (20E), which triggers larval molting and metamorphosis [12]. It is generally believed that the role of JH is to prevent premature metamorphosis, allowing larvae to undergo multiple rounds of molting until they reach an appropriate size [10]. This indicates that the regulation of developmental transitions in insects by ecdysteroids and JH is antagonistic. Farnesyl-pyrophosphate (FPP) is synthesized via the mevalonate pathway, after which JH is produced through the JH branch. FPP is synthesized from acetyl-CoA through a series of enzymatic reactions. Subsequently, FPP is converted into farnesoic acid by the actions of farnesyl pyrophosphatase and farnesol dehydrogenase. Finally, farnesoic acid is catalyzed by juvenile hormone acid methyltransferase (JHAMT) to form JHs [13]. JHAMT is the rate-limiting enzyme controlling JH synthesis. The *Jhamt* gene has been cloned in various insect species such as the *Bombyx mori* [14], *Drosophila melanogaster* [15], *Tribolium castaneum* [16] and *Aedes aegypti* [17].

Synthesized JH in the corpora allata is released into the hemolymph and transported via the hemolymph to various tissues. Among these, Hexamerin (HEX) is synthesized in the fat body and serves as one of the most crucial storage proteins in insects [18]. It is also involved in the transport of JH [19,20,21]. Research on the Western honey bee [22], the American cockroach [23], and termites [24] has demonstrated that JH can induce the expression of *Hex*. However, the main response gene of JH is *Kruppel homolog 1* (*Kr-h1*) [25]. A direct or indirect regulatory interplay exists between miRNAs and hormonal pathways in insects. 20-hydroxyecdysone (20E) or JH can induce or suppress the expression of let-7 [26,27]. Conversely, miRNAs can also indirectly modulate insect hormone production by targeting mRNA [28]. Studies have shown that the miRNA bantam in *Drosophila melanogaster* negatively regulates the expression of *Jhamt*, affecting larval metamorphosis [29]. miR-276 and miR-182013-5p influence metamorphosis and reproduction in migratory locusts by regulating *Jhamt* [30]. Spinetoram and cyantraniliprole disrupt JH synthesis by mediating the expression of the *farnesyl diphosphate synthase* gene (*FPPS*) and *Jhamt* through microRNA-9993/microRNA-2a-3p, leading to mortality in *Spodoptera frugiperda* [31]. Although the regulation of *Jhamt* by miRNAs has been reported in several insect species mentioned above, research on this interaction in honeybee (*Apis mellifera ligustica*) remains limited.

Honeybees, as one of the most important pollinating insects globally, play a crucial role in sustaining agricultural production, the survival and reproduction of wild plants, and maintaining ecosystem balance [32]. The miRNAs also serve important functions in regulating honeybee development. The miRNA Ame-bantam-3p targets the Multiple Epidermal Growth Factor-like Domains 8 gene to control larval–pupal development in *Apis mellifera* [33]. Ame-miR-980-3p targets *Atg2B* to participate in midgut remodeling [34]. Research indicates that JH and 20E interact with developmental genes and miRNAs to regulate pupal development in *Apis mellifera* [35]. Based on previously obtained transcriptomic data from the gut of *Apis mellifera ligustica* worker larvae [36], this study screened and identified the miRNA novel-m0027-3p in the larval gut. The expression profile of novel-m0027-3p was investigated during the larval and pupal stages. We focused on six target genes of novel-m0027-3p associated with insect hormone pathways. Among these, the target gene *AmJhamt*, as a rate-limiting enzyme in juvenile hormone synthesis, acts upstream in hormone biosynthesis. Furthermore, the regulatory relationship between novel-m0027-3p and its key target gene *AmJhamt* was validated, and its functional role in the larval development of *A. m. ligustica* was investigated. This work holds importance for elucidating how miRNAs modulate hormonal pathways to influence honeybee growth and development.

## 2. Materials and Methods

### 2.1. Rearing of Honeybee Larvae

The Western honeybee (*Apis mellifera ligustica*) colonies, preserved and maintained by the College of Bee Science at Fujian Agriculture and Forestry University, were reared and managed following standardized protocols. Five days after the queen lays eggs, combs containing larvae were transferred to a benchtop. Using a sterilized grafting needle, 2-day-old larvae were individually transferred into a 48-well plate at one larva per well, each well containing 50 µL of artificial diet (63% royal jelly, 30% sterilized water, 6% honey, and 1% yeast extract). The plates were then placed in a constant temperature and humidity chamber set at 35 ± 0.5 °C and 90% relative humidity, constant darkness. The artificial diet was replaced daily throughout the rearing period.

### 2.2. Stem-Loop RT-PCR

After hatching, 4- and 6-day-old larvae, 2-day-old prepupae, and pupae at 1, 3, 5, 7, and 9 days of age were collected (*n* = 3 per stage). Total RNA from each developmental stage was extracted using an RNA extraction kit (Accurate Biology, Changsha, China). The RNA was reverse-transcribed into cDNA with Hifair^®^ III 1st Strand cDNA Synthesis Kit (Yeasen, Shanghai, China) using stem-loop primers. PCR amplification was performed using 2 × Hieff^®^ PCR Master Mix (Yeasen, Shanghai, China) under the following program: initial denaturation at 94 °C for 5 min; 33 cycles of denaturation at 94 °C for 30 s, annealing at 56 °C for 30 s, and extension at 72 °C for 1 min; followed by a final extension at 72 °C for 10 min. The PCR products were analyzed by 2% agarose gel electrophoresis. A DNA fragment of approximately 100 bp was recovered, followed by TA cloning and Sanger sequencing. The sequences of the stem-loop RT-PCR primers are listed in Supplementary Table S1.

### 2.3. RT-qPCR

The cDNA synthesized in Section 2.2 was used as template for quantitative PCR (qPCR), which was performed using Hieff^®^ qPCR SYBR Green Master Mix (Yeasen, Shanghai, China) on a fully automated PCR analysis system (Tianlong, Xi’an, China). The thermal cycling protocol consisted of an initial denaturation step at 95 °C for 30 s, followed by 40 cycles of denaturation at 95 °C for 15 s and annealing/extension at 60 °C for 30 s. A melting curve analysis was subsequently conducted. The *U6* gene served as the internal control, and the primer sequences are listed in Supplementary Table S1. The relative expression level of novel-m0027-3p was calculated using the 2^−ΔΔCt^ method, with its expression level in 4-day-old larvae normalized to 1. The experiment included three biological replicates, each of which was assayed with three technical replicates.

### 2.4. Prediction and Functional Annotation of Target mRNAs

Following previously described methods [36,37], the target mRNAs of novel-m0027-3p were predicted using three software tools in combination: RNAhybrid version 2.1.2, miRanda version 3.3a, and TargetScan version 7.2. Default parameters were applied for all software. The intersection of the prediction results from the three tools was taken as the set of reliable target mRNAs. These target mRNAs were then aligned against the Gene Ontology (GO) database (http://geneontology.org/, accessed on 8 April 2025) and the KEGG database (https://www.kegg.jp/, accessed on 8 April 2025) using BLAST 2.16.0+ to obtain corresponding functional and pathway annotations. Charts and graphs were generated using the relevant tools available on the OmicShares cloud platform (www.omicshare.com, accessed on 10 April 2025).

### 2.5. Dual-Luciferase Reporter Assay

Based on the predicted targeting binding site (5′-CATCAATAAAACAT-3′) between novel-m0027-3p and *AmJhamt* (GenBank accession: NM_001327967.2), the corresponding binding site sequence (*AmJhamt-wt*) and its mutant sequence (*AmJhamt-mut*) were synthesized. These sequences were individually cloned into the pmirGLO vector (E1330, Promega, Madison, WI, USA). Bacterial cultures containing the respective monoclonal vectors were subjected to Sanger sequencing. For cultures with correctly aligned sequences, plasmids were extracted using an Endotoxin-Free Plasmid Extraction Kit (EM152-01, TransGen Biotech, Beijing, China). Based on the sequence of novel-m0027-3p, the mature novel-m0027-3p sequence was simulated as miRNA mimics (double-stranded), while the antisense strand of the novel-m0027-3p sequence was used as miRNA inhibitors (single-stranded). At the same time, their corresponding negative controls (Mimic-NC and Inhibitor-NC) were designed and chemically synthesized by Sangon Biotech (Sangon Biotech, Shanghai, China). All oligonucleotides were fully modified with 2′-O-methyl and phosphonothioates linkages to enhance stability and were HPLC-purified. The sequences are listed in Supplementary Table S2.

HEK-293T cells were maintained in DMEM (6125546, Gibco, Grand Island, NY, USA) supplemented with 10% fetal bovine serum (3168648, Gibco, Grand Island, NY, USA) and 1% penicillin–streptomycin (60162ES76, Yeasen, Shanghai, China), and cultured at 37 °C under 5% CO_2_ and 95% relative humidity. When cells reached approximately 90% density, they were seeded into 96-well plates and cultured for an additional 24 h to achieve 90–95% density. Transfection was performed using a lipid-based nucleic acid transfection reagent (40802ES03, Yeasen, Shanghai, China) according to the manufacturer’s instructions. Four transfection groups were set up as follows: 1: co-transfection of Mimic-NC and pmirGLO-*AmJhamt*-wt. 2: co-transfection of Mimics and pmirGLO-*AmJhamt*-wt. 3: co-transfection of Mimic-NC and pmirGLO-*AmJhamt*-mut. 4: co-transfection of Mimics and pmirGLO-*AmJhamt*-mut. Each well received 100 ng of plasmid and 10 pmol of either Mimics or Mimic-NC. All groups were performed in triplicate.

Twenty-four hours post-transfection, the culture medium in the plate was aspirated and discarded. Each well was then treated with 100 µL of cell lysis buffer and incubated on ice for 5 min. The lysate was subsequently transferred to RNase-free microcentrifuge tubes and centrifuged at 100× *g* for 1 min. A 20 µL aliquot of the supernatant was collected into a new RNase-free tube. Luciferase activity was measured sequentially using the Dual-Luciferase^®^ Reporter Assay System (11402ES60, Yeasen, Shanghai, China) according to the manufacturer’s instructions, by first adding the firefly luciferase reagent and then the *Renilla* luciferase reagent. Relative luciferase activity was calculated as the ratio of firefly luciferase activity to *Renilla* luciferase activity. Data from each group were normalized accordingly.

### 2.6. Overexpression and Knockdown of Novel-m0027-3p

Following a previously established method [38], 3-day-old larvae were individually fed 50 μL of artificial diet containing either Mimics, Inhibitors, Mimic-NC, or Inhibitor-NC, each at a final concentration of 40 pmol/g. The diet was replaced with a freshly prepared diet of the same composition every 24 h for three consecutive days. After the feeding period, the midguts of 4- to 6-day-old larvae were dissected. Total RNA was extracted, reverse-transcribed into cDNA, and subjected to qPCR analysis (as described in Section 2.3) to measure the relative expression levels of *AmJhamt*, *AmKr-h1*, and *AmHex70b*. The *Actin* gene served as the internal reference, and the primer sequences are provided in Supplementary Table S1.

### 2.7. JH Titer Assay

The 4- to 6-day-old larvae (*n* = 3 per time point) fed with Mimics, Inhibitors, Mimic-NC, or Inhibitor-NC were collected, rinsed three times with phosphate-buffered saline (PBS), and surface moisture was gently removed. An enzyme-linked immunosorbent assay (ELISA) kit (ml321040, mlbio, Shanghai, China) was used to detect juvenile hormone (JH) titer following the manufacturer’s instructions, with improvements made during JH extraction from larvae [39,40]. Briefly, each sample was homogenized in 200 μL of physiological saline on ice, followed by the addition of 1000 μL of n-hexane and 500 μL of 70% methanol. The mixture was centrifuged at 13,660× *g* for 10 min. The upper n-hexane layer was collected, and the solvent was removed using a freeze-dryer (ALPHAL-2, Christ, Osterode am Harz, Germany). Subsequently, 100 μL of standard diluent buffer (PBS buffer containing 1% bull serum albumin and 0.05% Tween-20) was added. 50 μL of the diluted standard and 50 μL of the sample were added to the reaction wells, respectively. Then, 50 μL of horseradish peroxidase (HRP)-labeled antibody was immediately added to allow the hormone to bind with the antibody, forming an antibody–antigen–enzyme-labeled antibody complex. After thorough washing, 3,3′,5,5′-tetramethylbenzidine (TMB) is added. Due to the catalytic action of HRP, TMB is initially converted into a blue product. Under acidic conditions, it turns into a yellow product. The intensity of the color is positively correlated with the insect hormone level in the sample. Absorbance (optical density, OD value) is measured at a wavelength of 450 nm using a microplate reader (Varioskan LUX, Thermos Fisher, Waltham, MA, USA). The assay was performed with three biological replicates, each consisting of three technical replicates.

### 2.8. Larval Body Weight

The 4- to 6-day-old larvae (*n* = 3 per group) fed with Mimics, Inhibitors, Mimic-NC, or Inhibitor-NC were collected, rinsed three times with PBS, and blotted dry. Individual larval body weight was then measured using an electronic balance (Sunny Hengping Instrument, Shanghai, China).

### 2.9. Data Analysis

GraphPad Prism version 8 (GraphPad, San Diego, CA, USA) was used for graph construction and statistical analysis. Data are presented as the mean ± SD. Statistical analysis was performed using Student’s *t*-test or one-way ANOVA, Tukey’s multiple comparisons. *, *p* < 0.05; **, *p* < 0.01; ***, *p* < 0.001; ns, non-significant.

## 3. Results

### 3.1. Identification and Target Analysis of Novel-m0027-3p

**Figure 1:**
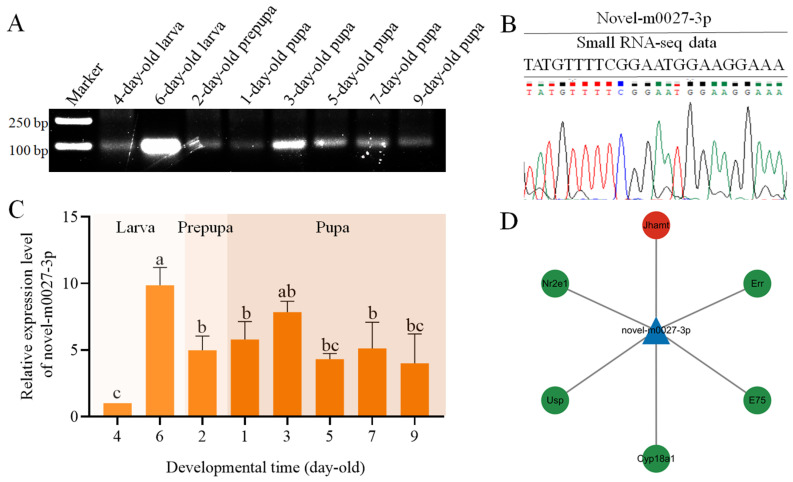
Identification and target analysis of novel-m0027-3p. (**A**) Stem-loop RT-PCR of novel-m0027-3p. (**B**) Sanger sequencing verification. (**C**) Expression profile of novel-m0027-3p from the larval to pupal stages detected by stem-loop RT-qPCR. The expression level of Novel-m0027-3p in 4-day-old larvae was normalized to “1”. One-way ANOVA, Tukey’s HSD test; different lowercase letters above the bars indicate significant differences (*p* < 0.05). (**D**) Hormone pathway-related target genes potentially regulated by novel-m0027-3p.

Stem-loop RT-PCR results showed that novel-m0027-3p could be detected across all eight time points examined, spanning the larval, prepupal, and pupal stages (Figure 1A). The sequence of novel-m0027-3p was further confirmed by Sanger sequencing (Figure 1B). The expression profile of novel-m0027-3p from the larval to the pupal stages was detected. The results showed that the relative expression level of novel-m0027-3p in 6-day-old larvae was significantly higher than that in other stages (*p* < 0.05). During the pupal stage, expression remained relatively stable overall, displaying an initial increase followed by a gradual decline. The lowest expression level was observed in 4-day-old larvae (Figure 1C).

Prediction of novel-m0027-3p target genes revealed that it potentially binds to 2109 mRNAs. Annotation against the GO database showed that these target mRNAs were associated with 15 biological processes, including cellular process, metabolic process, and single-organism process, as well as 10 molecular functions such as binding, catalytic activity, and molecular transducer activity (Figure S1). KEGG enrichment analysis identified target involvement in 10 metabolic pathways, including Glycolysis/Gluconeogenesis, Biosynthesis of amino acids, Inositol phosphate metabolism, and Citrate cycle (TCA cycle); the five genetic information processing pathways including RNA degradation, Nucleocytoplasmic transport, and RNA polymerase; the four environmental information processing pathways including Phosphatidylinositol signaling system, TGF-β signaling pathway, FoxO signaling pathway, and Hedgehog signaling pathway; the one cellular processes pathway (Endocytosis); and two organismal systems pathways (Dorso-ventral axis formation and Cytosolic DNA-sensing pathway) (Figure S2). We identified two hormone-related pathways (GO:0009755 and GO:0032870) through GO pathway enrichment, from which we obtained a total of six target genes associated with hormone synthesis or secretion. Subsequent research focused on *Jhamt* (*AmJhamt*), a key rate-limiting enzyme gene in JH synthesis in the *Apis mellifera*, while the other target genes were known as hormone pathway-related genes (Figure 1D).

### 3.2. Novel-m0027-3p Negatively Regulates the Expression of the Target Gene AmJhamt

**Figure 2:**
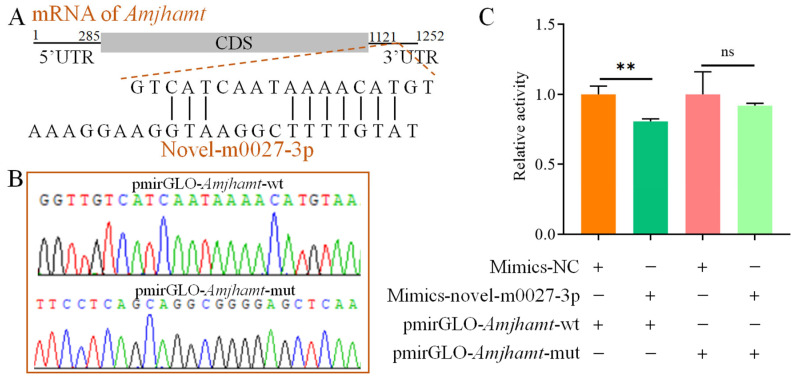
Validation of the targeting relationship between novel-m0027-3p and *AmJhamt*. (**A**) Schematic diagram of the predicted binding site of novel-m0027-3p on *AmJhamt*. (**B**) Sanger sequencing verification of the recombinant plasmids containing the wild-type (*AmJhamt-wt*) and mutant (*AmJhamt-mut*) binding sites. (**C**) A dual-luciferase reporter assay was performed to validate the interaction between novel-m0027-3p and *AmJhamt.* The Mimics-NC treatment relative activity was normalized to “1” in both the *AmJhamt-wt* and *AmJhamt-mut* groups. Student’s *t*-test; **, *p* < 0.01; ns, non-significant.

The predicted binding site for novel-m0027-3p on *AmJhamt* is located within the 3′ UTR, with a calculated binding free energy of −51.46 kJ/mol (Figure 2A). Recombinant plasmids for the dual-luciferase assay, pmirGLO-*AmJhamt*-wt and pmirGLO-*AmJhamt*-mut, were constructed (Figure 2B). The dual-luciferase assay showed that overexpression of novel-m0027-3p significantly suppressed luciferase activity compared with the control group (Mimic-NC) (*p* < 0.01). In contrast, this suppressive effect was abolished when the *AmJhamt* binding site was mutated, with no significant change in luciferase activity observed upon novel-m0027-3p overexpression (*p* > 0.05) (Figure 2C). These results indicate that novel-m0027-3p can directly bind to its target gene *AmJhamt*.

**Figure 3:**
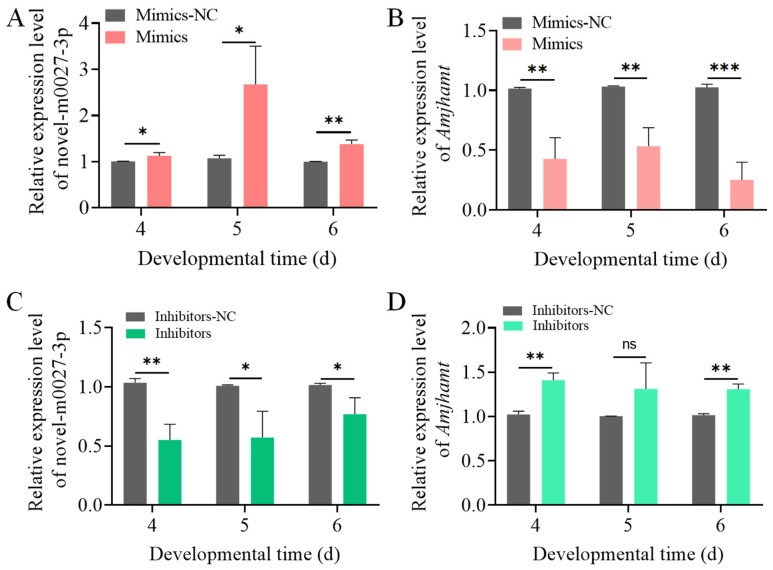
Validation of the regulatory between novel-m0027-3p and *AmJhamt*. (**A**,**C**) Relative expression levels of novel-m0027-3p following its overexpression or knockdown. (**B**,**D**) Relative expression levels of *AmJhamt* following overexpression or knockdown of novel-m0027-3p. Student’s *t*-test; *, *p* < 0.05; **, *p* < 0.01; ***, *p* < 0.001; ns, non-significant.

Given that the expression level of novel-m0027-3p was highest at the 6-day-old larvae, 3-day-old larvae were selected for feeding with either Mimics or Inhibitors to investigate the effect of novel-m0027-3p on 4 to 6-day-old larvae. After feeding Mimics, the relative expression level of novel-m0027-3p was significantly upregulated in 4 to 6-day-old larvae compared with the control group (*p* < 0.05), with an approximately 2.5-fold increase observed at 5-day-old larvae (Figure 3A). In contrast, the relative expression of its target gene, *AmJhamt*, was significantly downregulated across the same time points (*p* < 0.01) (Figure 3B). Similarly, after inhibition of novel-m0027-3p, its relative expression was significantly downregulated in 4 to 6-day-old larvae compared with the control (*p* < 0.05) (Figure 3C). The relative expression of *AmJhamt* was significantly upregulated at 4-day-old and 6-day-old larvae (*p* < 0.01), a non-significant increasing trend was observed at 5-day-old larvae (*p* > 0.05) (Figure 3D). These results demonstrate that novel-m0027-3p negatively regulates the expression of *AmJhamt* gene.

### 3.3. The Effects of Novel-m0027-3p on Hormone Signaling and Larval Body Weight

**Figure 4:**
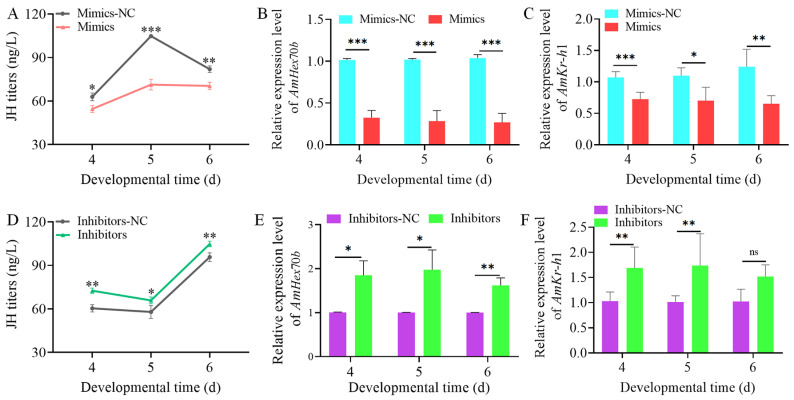
Effect of novel-m0027-3p on larval hormone signaling. (**A**,**D**) Changes in JH titer in 4- to 6-day-old larvae following overexpression or knockdown of novel-m0027-3p. (**B**,**E**) Relative expression levels of *AmHex70b* after overexpression or knockdown of novel-m0027-3p. Student’s *t*-test. (**C**,**F**) Relative expression levels of *AmKr-h1* after overexpression or knockdown of novel-m0027-3p. Student’s *t*-test; *, *p* < 0.05; **, *p* < 0.01; ***, *p* < 0.001; ns, non-significant.

Overexpression of novel-m0027-3p resulted in a significant reduction in the JH titers of 4 to 6-day-old larvae (*p* < 0.05) (Figure 4A). We subsequently assessed the expression of *AmHex70b* and *AmKr-h1*, the downstream gene in the JH signaling pathway. The decreased JH titer was accompanied by a marked downregulation in *AmHex70b* expression across the same developmental window (*p* < 0.01) (Figure 4B). Similarly, the relative expression of *AmKr-h1* was also significantly suppressed in the 4 to 6-day-old larvae (*p* < 0.01) (Figure 4C). In contrast, knockdown of novel-m0027-3p led to a significant increase in larval JH titer during 4–6 days of age (*p* < 0.05) (Figure 4D). Consistent with this change, the expression levels of both *AmHex70b* and *AmKr-h1* were significantly elevated over this period (*p* < 0.05) (Figure 4E,F).

**Figure 5:**
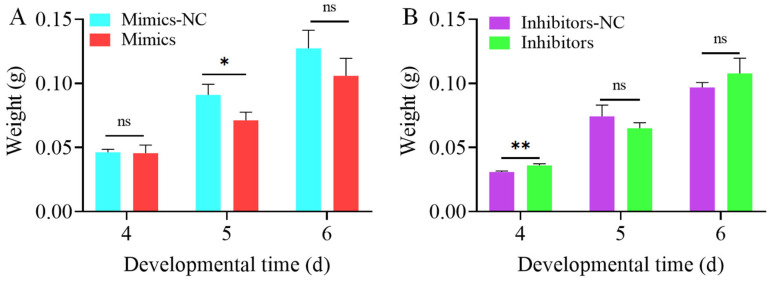
Effect of novel-m0027-3p on the larval body weight. (**A**) Changes in larval body weight after overexpression of novel-m0027-3p. (**B**) Changes in larval body weight after knockdown of novel-m0027-3p. Student’s *t*-test; *, *p* < 0.05; **, *p* < 0.01; ns, non-significant.

We next examined the impact of novel-m0027-3p on larval body weight. Following its overexpression, no significant difference in body weight of 4-day-old larvae was observed compared to the control group. The body weight of the 5-day-old larvae decreased significantly (*p* < 0.05), while a decreasing trend at 6-day-old did not reach statistical significance (*p* > 0.05) (Figure 5A). Conversely, knockdown of novel-m0027-3p led to a significant increase in body weight of 4-day-old larvae (*p* < 0.01). A slight decrease was observed but was not statistically significant at 5-day-old larvae (*p* > 0.05). Similarly, an increase in weight at 6-day-old also lacked statistical significance (*p* > 0.05) (Figure 5B). The results indicate that novel-m0027-3p has a limited effect on body weight.

## 4. Discussion

In the corpora allata of insects, JHAMT catalyzes the transfer of a methyl group from S-adenosyl-L-methionine to the carboxyl group of farnesoic acid or juvenile hormone acid, representing the final step in JH biosynthesis [14]. JH is a critical hormone for larval development in honeybees and plays a pivotal role in regulating caste differentiation [41,42]. Although several JH homologs exist in insects, such as JH 0, JH I, 4-methyl JH I, JH II, and JH III, only JH III has been isolated in *Apis mellifera* [43,44]. Based on previously obtained transcriptomic data from the larval guts of *A. mellifera* workers, this study screened and identified the miRNA novel-m0027-3p in the larval gut. Further overexpression and knockdown of novel-m0027-3p revealed that novel-m0027-3p affected the JH titers of larvae by regulating the expression of *AmJhamt*. These findings provide important insights into how miRNAs regulate hormone pathways to affect honey bee development.

Stem-loop RT-PCR results indicated that novel-m0027-3p is expressed throughout the developmental stages from larva to pupa in *A. mellifera* workers (Figure 1A), with its expression appearing highest in 6-day-old larvae. This observation was further confirmed by qPCR analysis (Figure 1C). During the pupal stage, the expression of novel-m0027-3p showed an overall pattern of an initial increase followed by a decline. These results suggest that novel-m0027-3p may play a significant role during the larval period and the larval–pupal metamorphic transition. The miRNAs primarily function by targeting mRNAs to regulate physiological processes [45]. Our prediction analysis revealed that novel-m0027-3p potentially interacts with 2109 mRNAs, although the actual binding relationships for these targets remain to be experimentally validated. GO and KEGG analyses of these target genes also indicated associations with the Wnt and Notch signaling pathways (Figures S1 and S2). Therefore, we focused specifically on hormone pathways relevant to honey bee larval development and identified 22 mRNAs, corresponding to six genes, that are potentially regulated by novel-m0027-3p, including *AmJhamt*, *AmE75*, and *AmUsp* (Figure 1D). Given the crucial role of JHAMT in JH synthesis, we subsequently investigated the regulatory relationship between novel-m0027-3p and *Jhamt*.

The prevailing view is that miRNAs primarily function as repressors by specifically binding to the 3′ UTR of mRNAs to inhibit gene expression [30,46]. Our dual-luciferase reporter assay demonstrated that novel-m0027-3p binds to the 3′ UTR of *AmJhamt* and negatively regulates its expression (Figure 2 and Figure 3). In *Locusta migratoria*, miR-276 has been shown to negatively regulate *Jhamt* expression, and knocking down *Jhamt* leads to premature molting, precocious metamorphosis, and impaired vitellogenesis in nymphs [30]. In *Drosophila melanogaster*, overexpression of miR-8 in the corpora allata increased the cell size of the gland and elevated the expression level of *Jhamt* [47]. Furthermore, the miR-9993/miR-2a-3p−FPPS/JHAMT−JH pathway mediates larval and pupal development in *Spodoptera frugiperda* [31]. JH is considered to play a key role in regulating the caste differentiation of social insects such as honeybees [41,42]. The regulatory effect of novel-m0027-3p on *AmJhamt* may influence JH titers, thereby impacting *Apis mellifera* caste differentiation. The ability of miRNAs to modulate gene expression subtly and efficiently makes them ideal candidates for regulating the complex developmental pathways that produce distinct phenotypes within a colony.

Previous studies have shown that overexpression and knockdown of miRNAs in honey bees can be achieved by feeding mimics or inhibitors [38]. Consistent with previous findings, our results demonstrate that upregulating or inhibiting the expression of novel-m0027-3p can modulate *AmJhamt* expression and affect JH titers in larvae. This, in turn, suppresses or promotes the expression levels of downstream JH-responsive genes, *AmHex70b* and *AmKr-h1* (Figure 4). Of note, the JH titers in the control groups (Mimic-NC and Inhibitor-NC) differed considerably, particularly in 5-day-old larvae. We speculate that this may be attributed to differences in the rearing environment between the two groups of larvae, such as temperature and humidity, leading to minor developmental variations. In contrast to the phenotypic responses to JH in other insects, *Apis mellifera* larvae are highly sensitive to JH between days 4 to 6, which is critical for caste differentiation [41,48]. The most important phenotypic indicators of caste differentiation are body weight and size. These findings align with our previous study showing that Ame-miR-2161 affects the larval-to-pupal transition in honey bees via the *AmJhamt* gene [49]. Our results suggested that novel-m0027-3p regulates *AmJhamt*, thereby affecting JH levels to a certain extent, but its impact on larval body weight is relatively limited. The potential hormone-related target genes bound by novel-m0027-3p are not limited to *AmJhamt* but may also include key hormonal signaling factors such as *AmE*75 and *AmUsp*. Therefore, the alteration in larval body weight induced by novel-m0027-3p may not be mediated solely through *AmJhamt*. Although novel-m0027-3p is predicted to target a large number of genes, this study primarily focused on *AmJhamt*. The regulatory roles of other predicted target genes remain important unresolved questions, representing a direction for future research.

In conclusion, this study confirms that novel-m0027-3p negatively regulates the expression of *AmJhamt*, and affects juvenile hormone biosynthesis in *Apis mellifera* larvae.

## Data Availability

The original contributions presented in this study are included in the article/Supplementary Material. Further inquiries can be directed to the corresponding authors.

## Supplementary Materials

The following supporting information can be downloaded at: https://www.mdpi.com/article/10.3390/insects17030288/s1, Figure S1: Loop graph of GO terms annotated by target mRNAs of novel-m0027-3p; Figure S2: Loop graph of KEGG pathways annotated by target mRNAs of novel-m0027-3p; Table S1: Primers for PCR and RT-qPCR; Table S2: Primers used for dual-luciferase assay.
