# Supplementary material for: MicroRNA Novel-m0027-3p Negatively Regulates Jhamt Gene and Affects Juvenile Hormone Biosynthesis in Apis mellifera Larvae
Source: Insects. 2026 Mar 6;17(3):288. doi: 10.3390/insects17030288 (PMC13026443; doi:10.3390/insects17030288)
Supplement: Supplementary file 1 [file insects-17-00288-s001.zip › insects-4159525-supplementary.pdf]

Supporting Information

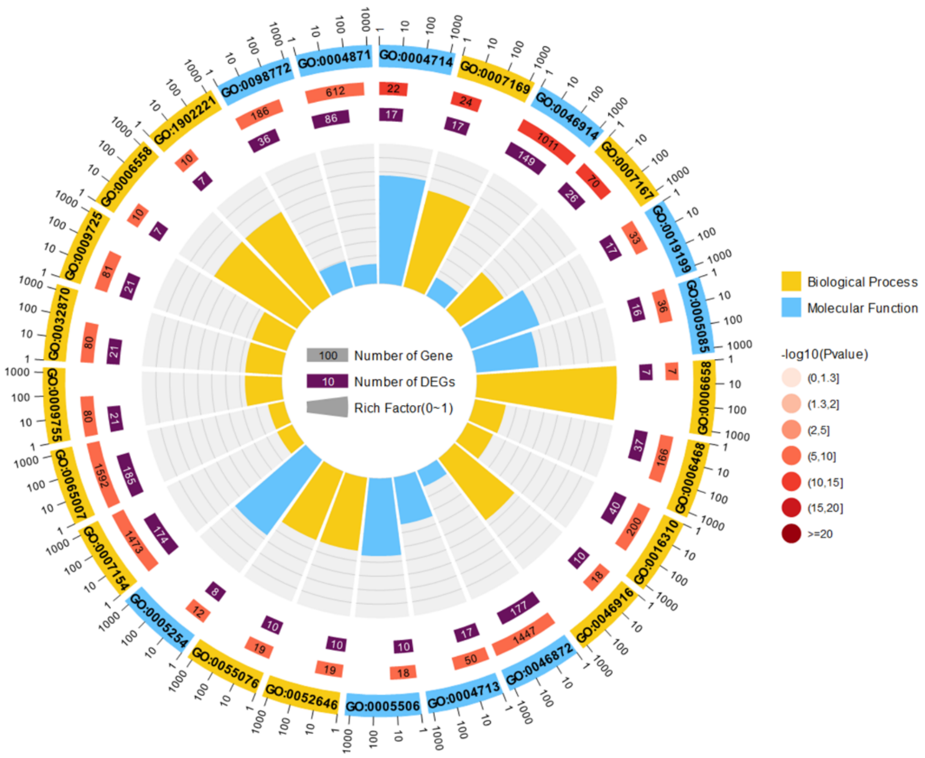

Figure S1. Loop graph of GO terms annotated by target mRNAs of novel-m0027-3p. GO:0098772, molecular function regulator; GO:0004871, signal transducer activity; GO:0004714, transmembrane receptor protein tyrosine kinase activity; GO:0007169, transmembrane receptor protein tyrosine kinase signaling pathway; GO:0046914, transition metal ion binding; GO:0007167, enzyme linked receptor protein signaling pathway; GO:0019199, transmembrane receptor protein kinase activity; GO:0005058, guanyl-nucleotide exchange factor activity; GO:0006658, Phosphatidylserine metabolic process; GO:0006468, protein phosphorylation; GO:0016310, phosphorylation; GO:0046916, cellular transition metal ion homeostasis; GO:0046872, metal ion binding; GO:0004713, protein tyrosine kinase activity; GO:0005506, iron ion binding; GO:0052646, alditol phosphate metabolic process; GO:005076, transition metal ion homeostasis; GO:005254, chloride channel activity; GO:0007154, cell communication; GO:0065007, biological regulation; GO:0009755, hormone-mediated signaling pathway; GO:0032870, cellular response to hormone stimulus; GO:0009725,cellular process; GO:0006558, L-phenylalanine metabolic process; GO:1902221, erythrose 4 phosphate/phosphoenolpyruvate family amino acid metabolic process;

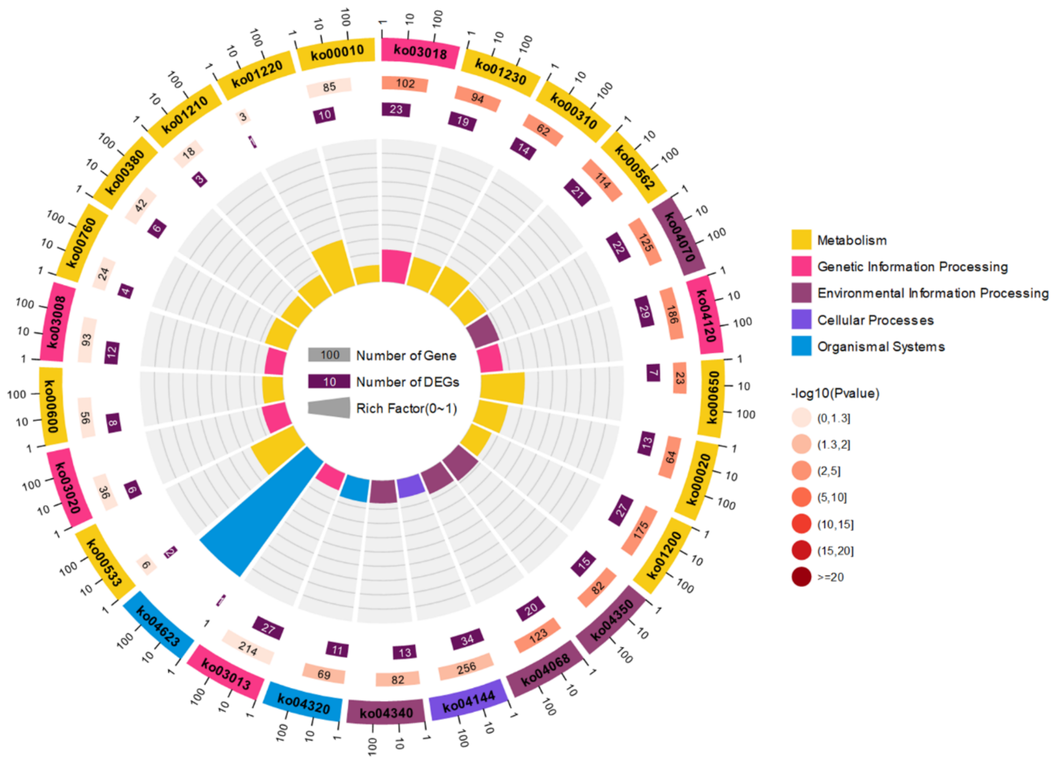

Figure S2. Loop graph of KEGG pathways annotated by target mRNAs of novel-m0027-3p. ko03018, RNA degradation; ko01230, Biosynthesis of amino acids; ko00310 ,Lysine degradation; ko00562, Inositol phosphate metabolism; ko04070, Phosphatidylinositol signaling system; ko04120, Ubiquitin mediated proteolysis; ko00650, Butanoate metabolism; ko00020, Citrate cycle (TCA cycle); ko01200, Carbon metabolism; ko04350, TGF-beta signaling pathway; ko04068, FoxO signaling pathway; ko04144, Endocytosis; ko04340, Hedgehog signaling pathway; ko04320, Dorso-ventral axis formation; ko03013, RNA transport; ko04623, Cytosolic DNA-sensing pathway; ko00533, Glycosaminoglycan biosynthesis - keratan sulfate; ko03020, RNA polymerase; ko00600, Sphingolipid metabolism; ko03008, Ribosome biogenesis in eukaryotes; ko00760, Nicotinate and nicotinamide metabolism; ko00380, Tryptophan metabolism; ko01210, 2-Oxocarboxylic acid metabolism; ko01220, Degradation of aromatic compounds; ko00010, Glycolysis/Gluconeogenesis;

Table S1. Primers for PCR and RT-qPCR

Primers	Sequence（5′-3′）	Purpose
novel-m0027-3p-loop	CTCAACTGGTGTCGTGGAGTCGGCA ATTCAGTTGAGCTTTCCTTC	Reverse transcription of miRNA into cDNA
novel-m0027-3p-loop-F	GCGTATGTTTTCGGAATGGA	PCR and qPCR of novel-m0027-3p
novel-m0027-3p-loop-R	AGTGCAGGGTCCGAGGTATT	
Amjhamt-F	TATGTATCACGACGAGGA	qPCR
Amjhamt-R	GAATGCTTTCTGGAAGTTT	

Hex70b-F	AACTCGCTCAACTTTCCACAA
Hex70b-R	GGCTCACATAACTAACCTCACC
*Kr-h*1-F	GCATTGGAAGCAGTTGAAGAAG
*Kr-h*1-R	GAAGGTACAGGACTCACAGGAT
actin-F	CCTAGCACCATCCACCATGAA
actin-R	GAAGCAAGAATTGACCCACCAA
U6-F	GTTAGGCTTTGACGATTTCG
U6-R	GGCATTTCTCCACCAGGTA

Table S2. Primers used for dual-luciferase assay

Primers	Sequence（5′-3′）	Purpose
Amjhamt-wt-F	CATATTTACCATGTTGGTTGTCATCAATAAAACATGTAAAACGC	For PCR of *Amjhamt* binding site wild type sequence
Amjhamt-wt-R	TCGAGCGTTTTACATGTTTTATTGATGACAACCAACATGGTAAATATGAGCT	
Amjhamt-wt-F	CGGAAAGGCAGTGGCGAGGCACCCAGGCTGTGATACGTAGAGC	For PCR of *Amjhamt* binding site mutation sequence
Amjhamt-wt-R	TCGAGCTCTACGTATCACAGCCTGGGTGCCTCGCCACTGCCTTTCCGAGCT	
mimic-novel-m0027-3p-sense	UAUCACAGCCAGCUUUGAUGAGC	PCR for synthesize the Mimics
mimic-novel-m0027-3p-antisense	UCCUUCCAUUCCGAAAACAUAUU	
mimic-NC-sense	UUCUCCGAACGUGUCACGUTT	PCR for synthesize the Mimics-NC
mimic-NC-antisense	ACGUGACACGUUCGGAGAATT	
inhibitor-novel-m0027-3p	UUUCCUUCCAUUCCGAAAACAUA	The Inhibitors
inhibitor-NC	CAGUACUUUUGUGUAGUACAA	The Inhibitors-NC
